# Patient complexity profiles in depression: a machine learning approach to personalized mental health

**DOI:** 10.3389/fpsyt.2026.1741860

**Published:** 2026-02-10

**Authors:** Paula Dagnino, Matias Salinas, Rodrigo Salas

**Affiliations:** 1 Facultad de Psicología y Humanidades, Universidad San Sebastián, Santiago, Chile; 2 Health Sciences and Engineering, Universidad de Valparaíso, Valparaíso, Chile; 3 Biomedical Engineering School, Faculty of Engineering, Universidad de Valparaíso, Valparaíso, Chile; 4 Center of Interdisciplinary Biomedical and Engineering Research for Health (MEDING), Valparaíso, Chile; 5 Millennium Institute for Intelligent Healthcare Engineering (iHealth), Santiago, Chile

**Keywords:** depression complexity profiles, machine learning, personalized mental health treatment, explainable AI for mental health, profile-based interventions

## Abstract

**Background:**

Patient complexity in mental health varies substantially, yet treatment approaches often rely on standardized protocols. Identifying distinct complexity profiles may support stratified care and more personalized intervention planning in depression.

**Objective:**

To identify distinct patient complexity profiles in depression across sociodemographic, clinical, and psychosocial indicators and to evaluate their clinical relevance for personalized treatment planning.

**Methods:**

We analyzed complete-case data from 270 adults with major depression using a Knowledge Discovery in Databases framework. Twelve indicators were analyzed via Principal Component Analysis followed by K-means clustering. Robustness was evaluated using supervised validation with a Random Forest classifier and SHAP-based interpretability analysis. Between-profile comparisons were conducted, and expert clinicians evaluated clinical relevance.

**Results:**

Model selection supported a three-cluster solution (k = 3: Low-, Moderate-, and High-complexity profiles). The solution was validated using a Random Forest classifier with strong performance (accuracy = 0.91). Statistical comparisons showed that the Low-complexity profile (n = 100, 37.0%) was older and more often partnered and employed, with lower depressive symptoms and better personality functioning. The Moderate-complexity profile (n = 87, 32.2%) was younger, predominantly unpartnered, and had the lowest employment rate and medical comorbidity. The High-complexity profile (n = 83, 30.7%) showed the most severe presentation, characterized by higher depressive symptoms, greater childhood maltreatment, and impaired personality functioning. Clinical experts confirmed interpretability and suggested tailored strategies for each profile.

**Conclusions:**

Machine learning identified clinically meaningful patient complexity profiles with significant differences across multiple domains. These profiles provide a framework for stratified care and personalized intervention planning, moving beyond one-size-fits-all approaches.

## 1 Introduction

The field of mental health increasingly recognizes that patients seeking treatment comprise a heterogeneous population whose complexity extends beyond diagnostic categories (1–3). Patient complexity encompasses the multifaceted interplay of sociodemographic, clinical, and psychosocial factors that influence treatment response, functional outcomes, and healthcare utilization (4, 5). Rather than viewing complexity as a barrier to care, emerging evidence suggests that systematic stratification of complexity profiles can enable more personalized and effective treatment approaches (2, 6, 7).

Depression is a fundamentally heterogeneous condition, conceptualized as a collection of distinct phenotypes with different underlying mechanisms, clinical presentations, and treatment responses (6, 8). This heterogeneity is evident at multiple levels. At the symptom level, DSM criteria permit 227 unique symptom combinations that meet diagnostic thresholds (9). At neurobiological and genetic levels, recent frameworks propose specific depression subtypes and demonstrate heterogeneity across subgroups (10, 11).

The concept of patient complexity moves beyond the traditional biomedical model by incorporating psychological, social, and environmental dimensions (7, 12, 13). The Vector Model of Complexity proposed by Safford (14) emphasizes the interplay of biological, socioeconomic, cultural, environmental, and behavioral determinants as contributors to complexity. In mental health care, clinicians often intuitively adapt interventions to the unique circumstances of each patient (12), yet empirical knowledge about how complexity is composed and how it can be stratified in depression remains limited.

Complexity in depression arises from the confluence of sociodemographic, clinical, and psychosocial indicators that are often studied in isolation but rarely examined as integrated, multidimensional patterns. Sociodemographic factors such as age (2, 15), gender (2, 15, 16), relationship status (17), educational attainment (17–20), and employment status (2, 21, 22) are associated with depression severity and outcomes, and data-driven work suggests these factors can concentrate with clinical burden in specific subgroups (22, 23). Clinical indicators such as medical comorbidity can complicate both depression management and the care of co-occurring conditions through bidirectional relationships (24). Psychosocial indicators—including impairments in personality functioning, emotional dysregulation, and adverse childhood experiences—are consistently linked to greater severity, chronicity, and treatment complexity (25–36).

Despite the established relationship between these indicators and depression outcomes, much of the literature has focused on bivariate associations rather than multidimensional configurations that define distinct patient subgroups. In addition, many complexity models were developed in general medicine and have limited validation in psychiatric populations, while routine clinical settings often lack standardized, reproducible complexity stratification methods beyond clinical judgment (12).

By examining depression through a multidimensional lens that integrates sociodemographic, clinical, and psychosocial indicators, this study characterizes how these factors combine into distinct patient complexity profiles. The present study aimed to: (1) determine whether distinct patient complexity profiles exist in a treatment-seeking depression sample; (2) comprehensively characterize the identified profiles across interrelated domains; (3) identify which specific indicators most strongly differentiate complexity levels; and (4) assess whether mental health professionals recognize the profiles as clinically meaningful and can suggest tailored interventions.

Based on existing literature on patient complexity and depression heterogeneity, we hypothesized that: (H1) distinct complexity profiles would emerge in this treatment-seeking depression population, representing qualitatively different patterns of sociodemographic, clinical, and psychosocial indicators; (H2) profiles would show significant differences across demographic factors, clinical severity, personality functioning, emotional dysregulation, and trauma history; and (H3) higher complexity would be characterized by a greater accumulation and co-occurrence of risk factors across multiple domains rather than elevation in any single factor.

## 2 Materials and methods

### 2.1 Participants and procedure

At one outpatient clinic, adults aged 18 years or older seeking mental health care underwent a comprehensive clinical diagnostic interview with a psychiatrist or psychologist to confirm the presence of a major depressive episode according to the ICD-10 (World Health Organization, 1992). Those who agreed to participate in the study signed an informed consent form, ensuring confidentiality and voluntary participation. All participants were treatment-seeking individuals presenting for their first psychological consultation at the clinic. The research protocol was approved by the Universidad San Sebastián Ethics Committee (Protocol #151-22) and conducted in accordance with the Declaration of Helsinki.

We excluded patients with (1) primary substance use disorders, (2) active psychotic symptoms, (3) significant cognitive impairment, (4) primary eating disorders, or (5) acute suicidal risk requiring immediate intervention. These exclusions were determined through initial diagnostic interviews and were intended to focus the sample on patients suitable for outpatient psychotherapy.

Participants completed sociodemographic questionnaires and the Patient Health Questionnaire-9 (PHQ-9) before their first psychological consultation. Additional instruments—including the Childhood Trauma Questionnaire-Short Form (CTQ-SF), Operationalized Psychodynamic Diagnostics Self-Rating Questionnaire Short-Form (OPD-SQS), and Difficulties in Emotion Regulation Scale (DERS)—were completed at home and returned at the beginning of the second consultation due to their length.

The sample consisted of 270 adults (see **Table 1**), of whom 69.3% were female and 30.7% were male. The mean age was 35.2 years (SD = 13.0, range 18-65). Educational attainment was as follows: 27.4% had basic or secondary education, 65.2% had higher education, and 7.4% had a specialization or postgraduate degree. Employment status was distributed as 48.9% employed, 51.1% unemployed. Regarding relationship status, 39.9% of participants were in a relationship, while 60.7% were not. Previous psychological treatment was reported by 23.7% of participants, family history of psychological disorders by 58.9%, and medical comorbidities by 78.9%.

**Table 1:** Sample demographics and clinical characteristics with descriptive statistics.

Variable	Category	Total sample (N = 270)
Age, years		35.2 (13.0)
Sex	Male	83 (30.7%)
	Female	187 (69.3%)
Marital Status	Partnered	106 (39.3%)
	Unpartnered	164 (60.7%)
Education level	Basic/secondary	74 (27.4%)
	Higher education	176 (65.2%)
	Graduate/postgraduate	20 (7.4%)
Employment status	Employed	132 (48.9%)
	Unemployed	138 (51.1%)
Previous psychological treatment	Yes	64 (23.7%)
	No	206 (76.3%)
Family psychiatric history	Yes	159 (58.9%)
	No	111 (41.1%)
Medical comorbidity	Yes	213 (78.9%)
	No	57 (21.1%)
		M ± SD
Depressive symptoms (PHQ-9)		16.38 (6.6)
Childhood Maltreatment (CTQ)		50.9 (18.5)
Personality functioning (OPD-SQS)		27.0 (10.76)
Emotional dysregulation (DERS)		75.84 (21.0)

Codes were transformed for clarity: Sex: 1 = Male, 2 = Female. Marital status: 1 = Partnered, 2 = Unpartnered. Education: 1 = Basic/secondary, 2 = Higher education, 3 = Graduate/postgraduate. Employment: 1 = Employed, 2 = Unemployed. Medical comorbidity: 1 = Yes, 2 = No. Family psychiatric history: 1 = Yes, 2 = No. Previous psychological treatment: 1 = Yes, 2 = No.

Clinical characteristics indicated moderate to severe depression severity (PHQ-9: M = 16.38, SD = 6.6), significant difficulties with emotion regulation (DERS: M = 75.84, SD = 21.0), moderate childhood trauma exposure (CTQ: M = 50.9, SD = 18.5), and mild to moderate impairment in personality functioning (OPD: M = 27.0, SD = 10.76).

### 2.2 Measures

For reporting purposes, variables were grouped into sociodemographic (e.g., age, marital status, employment), clinical (e.g., depressive symptomatology, medical comorbidity), and psychosocial indicators (e.g., childhood trauma, emotion regulation, personality functioning). However, in line with contemporary complexity models, these domains were conceptualized as interdependent contributors to overall patient complexity rather than isolated categories.

Sociodemographic Data were collected using a self-report form. It assessed age, gender. Marital status was categorized as partnered (married or cohabiting) and unpartnered (single, separated, divorced, or widowed). This classification focuses on the presence of a stable cohabiting partner as a proxy for social support. Employment status (employed vs. unemployed), educational level (basic/secondary, higher education, or specialization/postgraduate), previous psychological treatment (yes/no), family psychiatric history was assessed as a dichotomous variable (yes, presence or no, absence of psychiatric disorders in first-degree relatives, and medical comorbidity (yes/no).

Depressive symptoms: The PHQ-9 (37) is a widely used nine-item questionnaire based on DSM-IV criteria for major depression. Items are rated on a 4-point Likert scale (0 = “not at all” to 3 = “nearly every day”), with total scores ranging from 0 to 27. Higher scores indicate greater depression severity. The Spanish version validated in Chilean primary care populations was used (38). The cut-off score is 16.

Childhood Maltreatment: The CTQ-SF (39) is a 28-item retrospective self-report instrument assessing five types of childhood maltreatment: physical abuse, emotional abuse, sexual abuse, physical neglect, and emotional neglect. Items are rated on a 5-point Likert scale (from 1, ‘never’ to 5, ´always’). In the present study, only the CTQ-SF total score was used as an indicator of overall childhood trauma exposure; the five subscale scores were not analyzed separately. The CTQ total score was calculated as the sum of the five subscales (emotional abuse, physical abuse, sexual abuse, emotional neglect, and emotional neglect), ranging from 25 to 125. The three validity items (items 10, 16, 28) were not included in the total score calculation. Higher scores indicating greater exposure to childhood trauma. The Chilean validation by (40) was used.

Emotion Regulation: The DERS (41) is a 36-item self-report questionnaire measuring difficulties in emotion regulation across six dimensions: lack of emotional awareness, lack of emotional clarity, non-acceptance of emotions, limited access to regulation strategies, impulse control difficulties, and difficulties with goal-directed behavior. Items are rated on a 5-point scale (1 = “almost never” to 5 = “almost always”), with total scores ranging from 36 to 180. Higher scores indicate greater difficulties with emotion regulation. The Chilean adaptation by Guzmán- (42) was used. The cut-off score is 73.

Personality Functioning: The OPD-SQS (43) assesses dimensional vulnerabilities in personality functioning across three domains: self-perception, interpersonal contact, and relationship patterns. The 12-item short form uses a 5-point Likert scale (0 = “not true at all” to 4 = “completely true”), with total scores ranging from 0 to 48. Higher scores indicate greater impairment in personality functioning. The 12-item short form is currently undergoing validation in Chilean samples, with the full version demonstrating adequate psychometric properties in prior work (44, 45).

### 2.3 Statistical analysis

**Figure 1:**
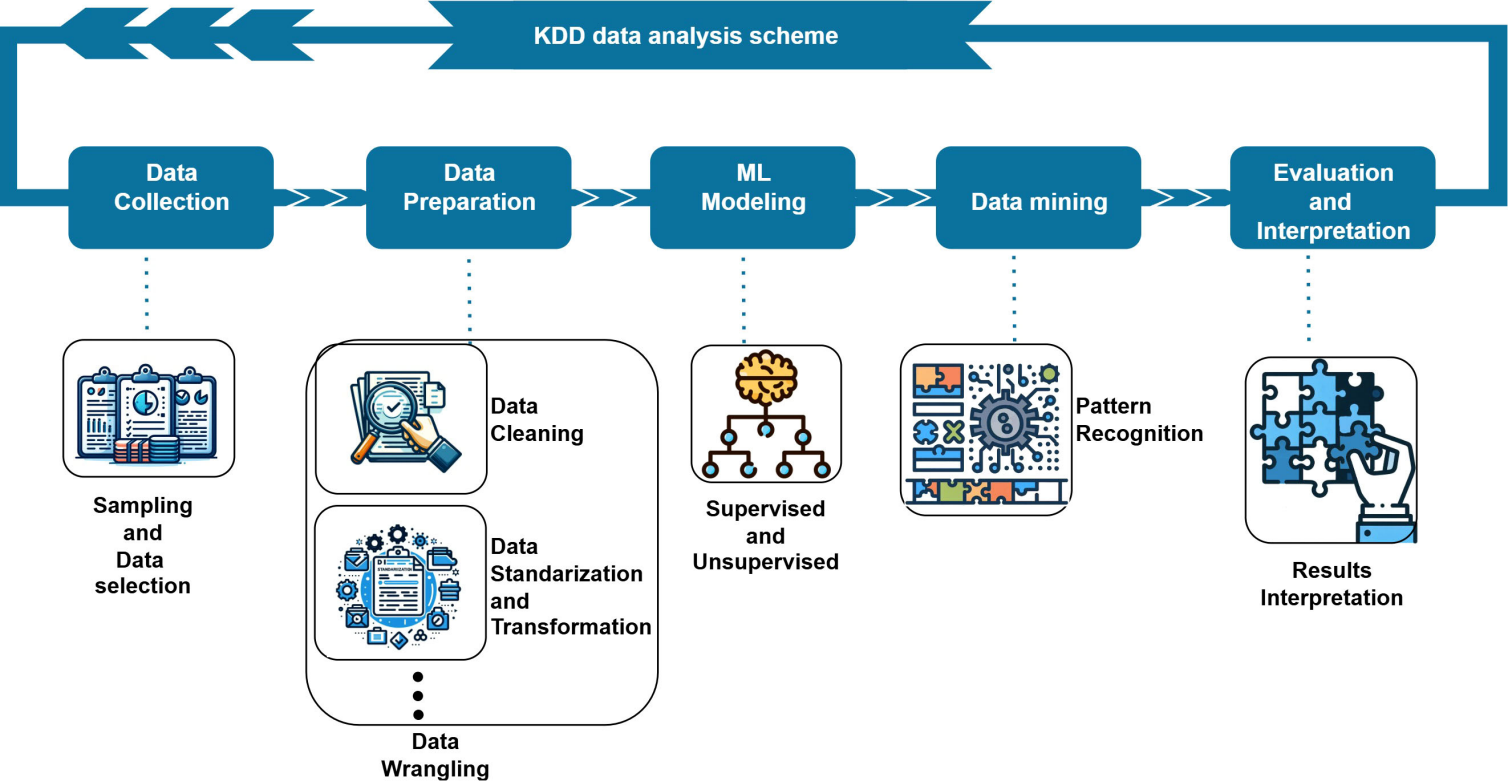
Knowledge Discovery in Databases (KDD) framework used to identify depression complexity profiles.

The analytical pipeline followed the five stages of the Knowledge Discovery in Databases (KDD) framework, (selection, preprocessing, transformation, data mining, and evaluation), as illustrated in **Figure 1**. Expert validation was also included to ensure clinical relevance and practical applicability (see **Figure 1**).

Data Preprocessing: Data were imported from an Excel database and non-numeric placeholders (e.g., “?”) were recoded as missing values (NaN). An identifier variable was removed prior to analysis. Variables were selected a prior to analysis based on clinical relevance. Information available in the database was incorporated through the corresponding total scores of each instrument, which were treated as composite variables representing the associated constructs, rather than considering individual questionnaire items separately. Based on this selection process, a predefined set of 12 clinically relevant variables was retained for analysis, encompassing sociodemographic, clinical, and psychosocial measures.

No statistical imputation was applied. Complete-case filtering was used to ensure consistency of the multivariate matrix, resulting in the exclusion of two participants with missing values in at least one selected variable. Therefore, the final sample consisted of 270 patients. Continuous variables were standardized using z-score normalization (mean = 0, SD = 1) prior to dimensionality reduction and clustering, while categorical variables were retained in their original coding for descriptive and interpretive purposes.

Dimensionality Reduction: Principal Component Analysis (PCA) was applied to the standardized variables (z-scores) to reduce dimensionality while preserving most of the variance. We retained the leading components that captured the main data structure and used their scores for 3D visualization and clustering. Loadings were interpreted as the correlation between each variable and each component, their magnitude indicated the variable’s contribution to each axis, and their sign indicated the direction of the relationship.

Clustering Analysis: K-means clustering was selected as the primary unsupervised learning technique due to its effectiveness with continuous variables and its ability to create compact, spherical clusters. The optimal number of clusters was determined through a systematic evaluation using multiple validation metrics, including the silhouette coefficient, Calinski-Harabasz index, and Davies-Bouldin index.

Supervised Validation: The robustness of clustering was evaluated using supervised learning approaches. A Random Forest classifier was trained to predict cluster membership based on the original features. High predictive accuracy indicated that the clusters reflected genuine data patterns rather than algorithmic artifacts.

Feature Interpretation: SHAP (SHapley Additive exPlanations) analysis quantified the individual contributions of variables to cluster assignment. This approach provided transparent explanations of how each feature influenced model decisions, enabling the translation of statistical patterns into clinically meaningful insights.

Statistical Comparisons: Comprehensive statistical testing identified significant differences between clusters across demographic and clinical variables. Chi-square tests assessed the distributions of categorical variables, with the Bonferroni correction applied for multiple comparisons, and effect sizes were quantified using Cramer’s V to estimate the magnitude of associations.

One-way ANOVA tested group differences for continuous variables, followed by Tukey’s HSD *post hoc* tests for specific pairwise comparisons, with effect sizes reported as η² to quantify the proportion of variance explained by cluster membership.

Expert Validation: Clinical descriptions of each patient profile were submitted to mental health experts for validation of clinical coherence, face validity, and therapeutic recommendations. Experts evaluated whether these profiles aligned with established clinical knowledge and provided treatment recommendations for each profile.

Software: Analyses were conducted using Python 3.9 with the scikit-learn, pandas, numpy, and shap libraries. R 4.3 was used for supplementary statistical analyses. Statistical significance was set at α = 0.05 for all tests.

## 3 Results

An unsupervised Knowledge Discovery in Databases (KDD) methodology was employed, beginning with preprocessing of the dataset containing 12 key sociodemographic, and clinical variables. Dimensionality reduction and clustering analyses were then conducted to identify homogeneous patient groups.

### 3.1 Dimensionality reduction and clustering solution

Principal Component Analysis (PCA) was applied, successfully transforming the original variables into a three-dimensional feature space (PC1−PC3). These principal components captured the data’s most significant variance and patterns, allowing for a simplified, interpretable 3D representation. K-means clustering was then used to partition the sample into internally homogeneous groups.

**Figure 2:**
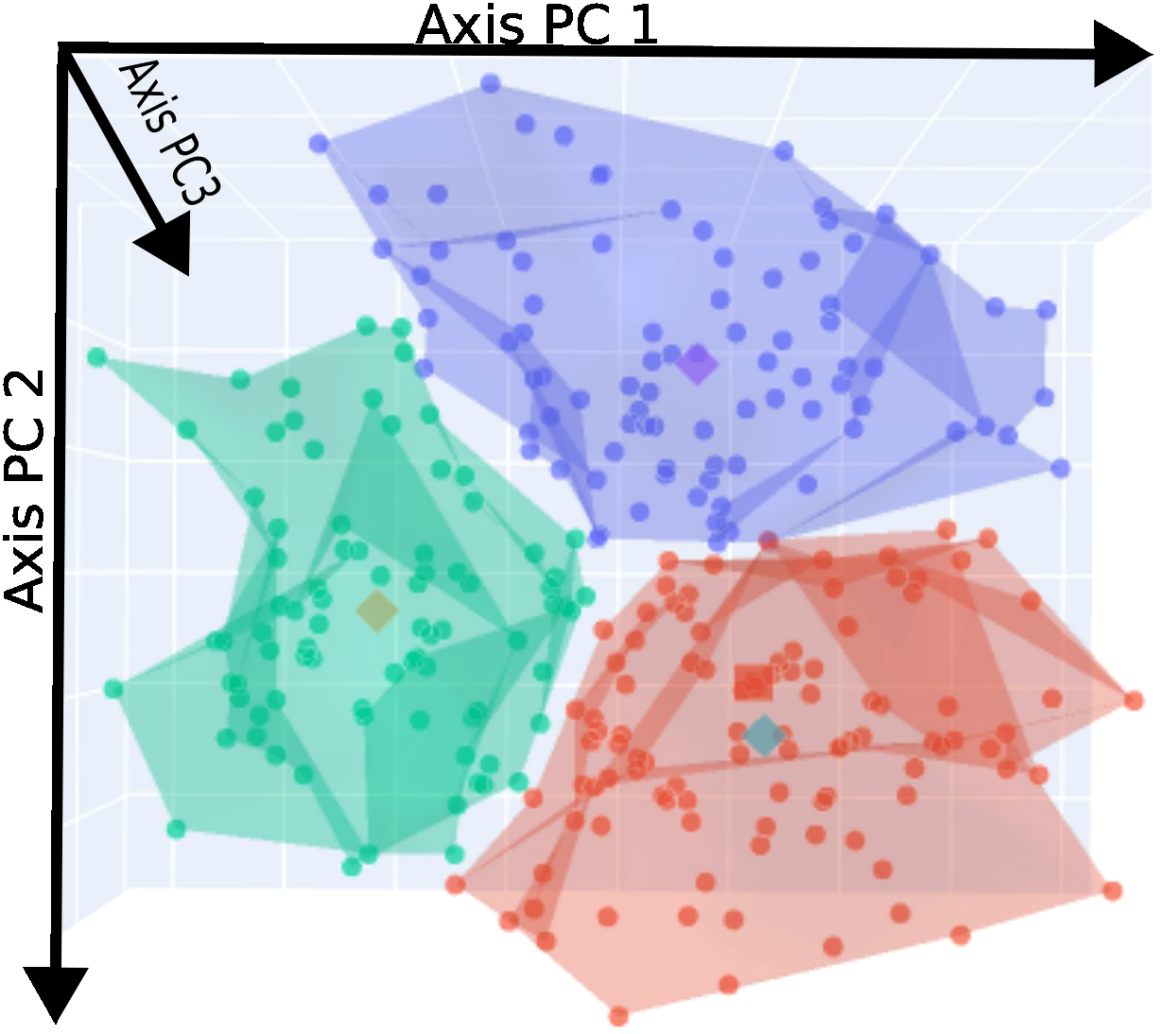
3D PCA projection of participants colored by risk clusters.

Model selection favored a three-cluster solution (k = 3), which was strongly supported by multiple clustering indices. The Silhouette index increased from 0.2707 (k = 2) to 0.2962 (k = 3), indicating improved cluster separation and internal cohesion. The Calinski-Harabasz index was higher for the three-cluster model (127.0177), reflecting greater between-group dispersion. Additionally, the Davies-Bouldin index decreased from 1.4188 (k = 2) to 1.1475 (k = 3), suggesting tighter and better-separated clusters. The Dunn index showed comparable values for the three- and four-cluster solutions, supporting the robustness of the three-cluster model. Visual inspection of the three-dimensional PCA space (**Figure 2**) further indicated clear spatial separation between clusters (red: Cluster 1; green: Cluster 2; blue: Cluster 3).

### 3.2 Cluster identification and description

**Figure 3:**
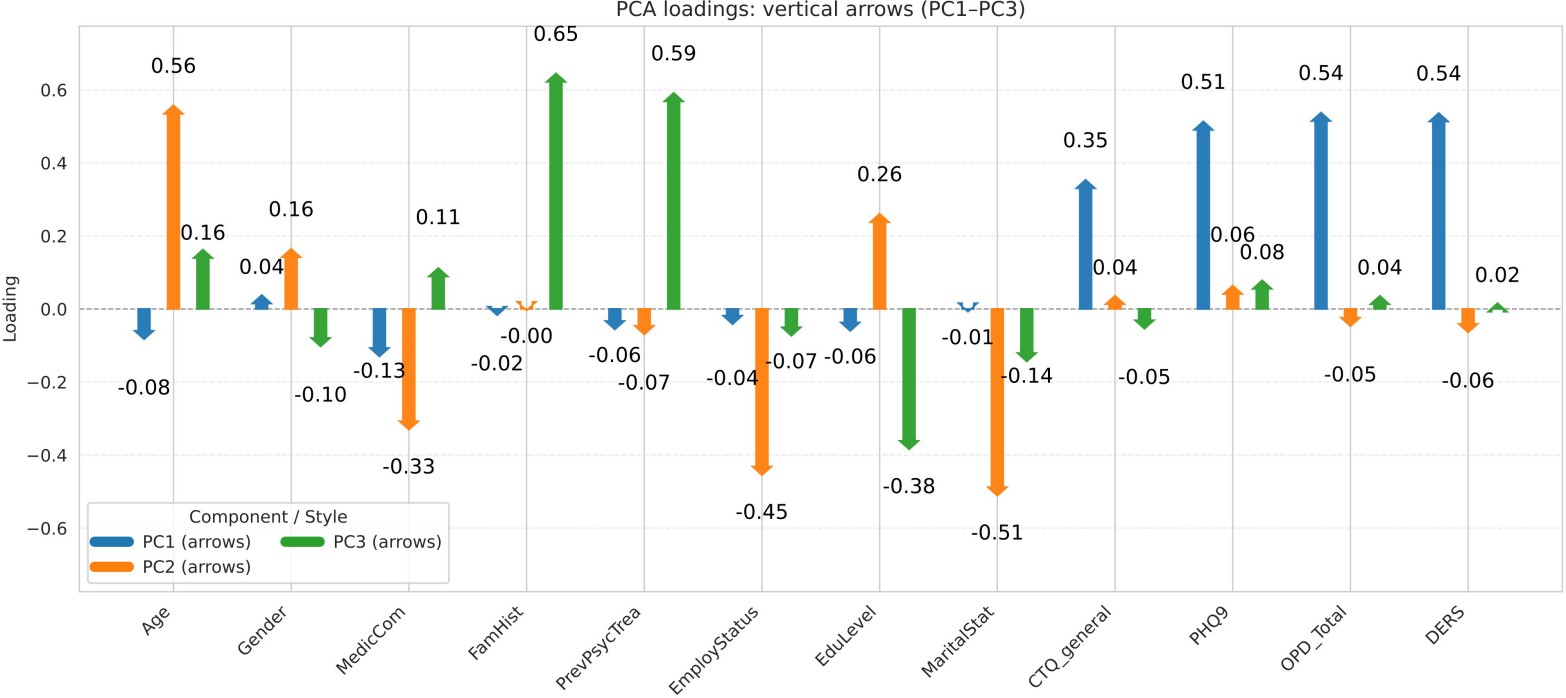
PCA loadings illustrating variable weights in cluster separation space.

To better understand which original variables contributed most to the formation of these clusters, we analyzed the PCA loadings (**Figure 3**). The loading vectors provide important insights into the variables that most strongly drive cluster heterogeneity. Variables with longer vectors and vectors pointing in similar directions are more strongly correlated, while those pointing in opposite directions are negatively associated.

In simpler terms, the first three principal components can be interpreted as new coordinate axes that summarize the information from all original variables. As shown in **Figure 3**, the PC1 axis is primarily influenced by clinical indicators such as depressive symptom, personality functioning, childhood maltreatment and emotion regulation. The PC2 axis represents more sociodemographic factors, including age and clinical medical comorbidities. The PC3 axis incorporates elements related to family members with psychiatric problems and previous psychological treatment. It also reflects additional clinical variables, such as depressive symptoms, childhood maltreatment, personality functioning, and emotional dysregulation.

When these axes are projected into the 3D PCA space (**Figure 2**), the three clusters show a clear spatial separation, highlighting the robustness and interpretability of the clustering solution.

### 3.3 Supervised confirmation

In addition to the unsupervised analysis, we trained a supervised classification model to predict cluster membership using the selected features. A Random Forest classifier was chosen. The model was trained using a stratified train–test split (80% training, 20% testing) to preserve the proportional representation of the three clusters, with hyperparameters optimized via cross-validation. The final model consisted of 150 trees, a maximum tree depth of 5, and 5 features considered at each split, with balanced class weights applied to account for cluster size differences.

The classifier demonstrated strong and stable performance, with accuracy, precision, sensitivity, and F1-score all reaching 0.91 ± 0.01, while the error rate remained low (0.11 ± 0.01), indicating robust classification capabilities. These results suggest that the selected features retained sufficient discriminative power to reliably distinguish cluster membership.

**Figure 4:**
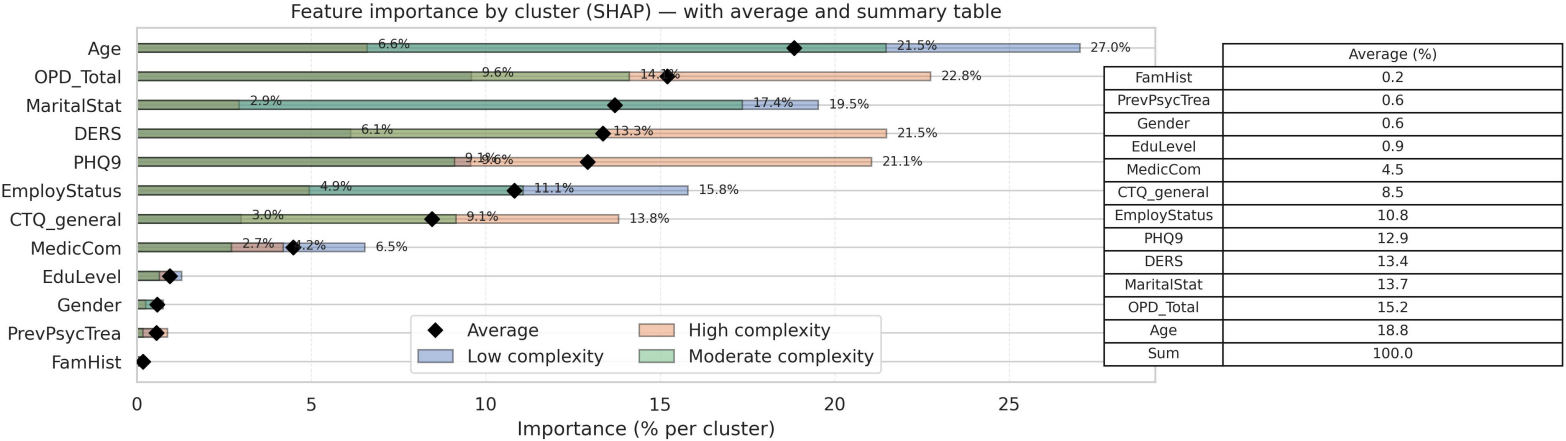
Global SHAP importance ranking.

To improve model transparency and better understand the influence of individual features, SHAP values were calculated. This specific SHAP analysis (see Appendix) provided a ranking of features based on their average impact on model predictions across all instances (see **Figure 4**). The presence and intensity of each feature within the clusters enabled us to identify and label the three profiles according to their overall level of complexity: low-complexity (L), moderate-complexity (M), and high-complexity (H) profiles.

The global SHAP analysis ranked features by their average impact on model predictions across all instances (see **Figure 4**). The global feature importance rankings were: (1) age (18.7%, 2) personality functioning (15.7%, 3) marital status (13.7%, 4) emotional regulation (13.4%, 5) depressive symptomatology (12.9%), and (6) employment status (10.8%). Together, these six features accounted for approximately 85% of the model’s predictive power.

**Figure 5:**
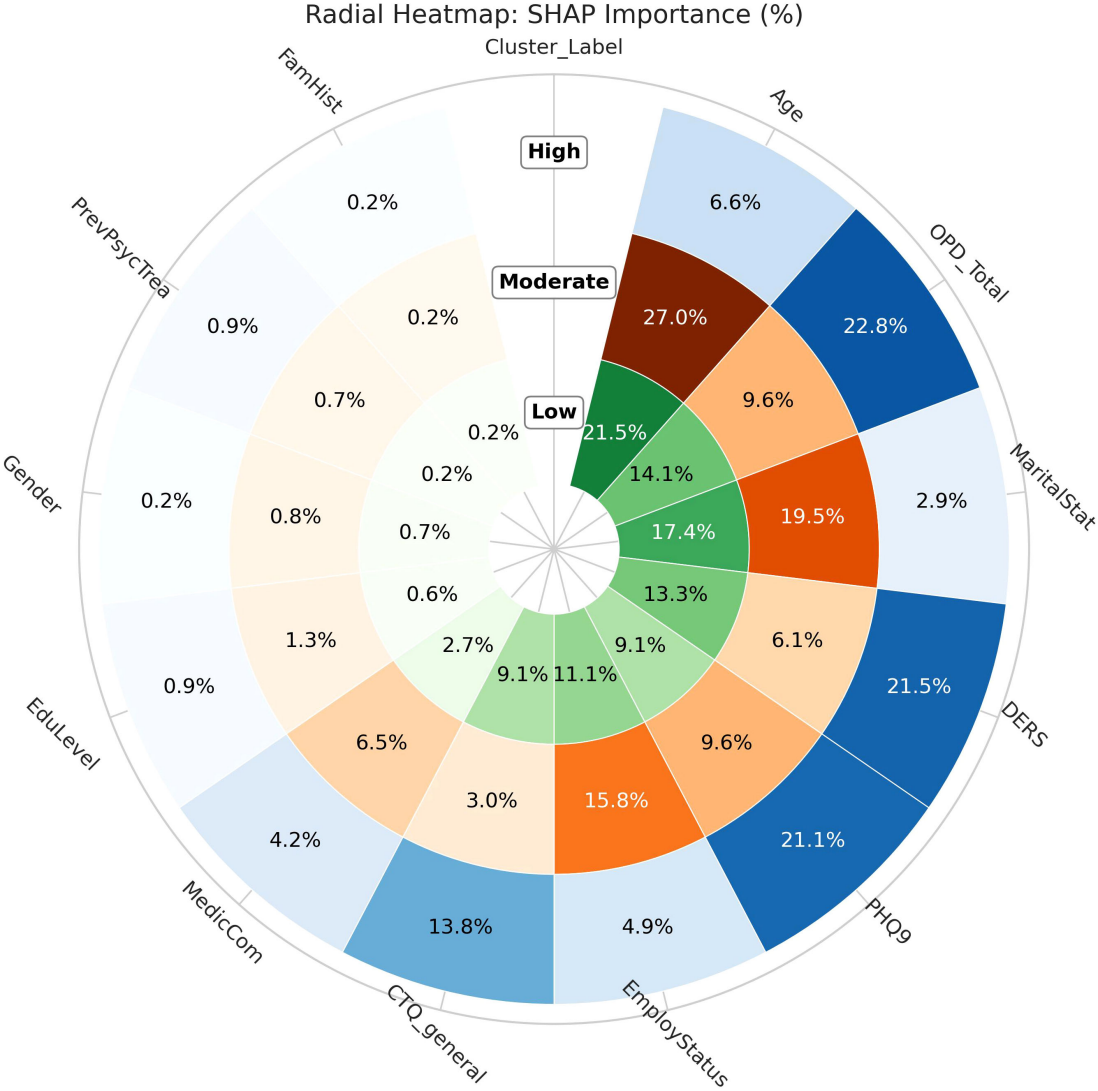
Cluster-specific SHAP importance across risk profiles.

Beyond the global ranking of features, the cluster-specific SHAP analysis revealed that the model relies on different feature sets across the complexity profiles (**Figure 5**). Predictions for the high-complexity profile were primarily driven by a compact set of variables—depressive symptomatology, personality functioning, emotional regulation, and childhood maltreatment—accounting for most of the predictive contribution. In contrast, the moderate-complexity profile relied on a broader combination of sociodemographic (age, marital status, employment) and clinical features, suggesting a more heterogeneous classification rule. The low-complexity profile showed the most distributed pattern, with the model integrating multiple contextual and clinical dimensions without a single dominant feature.

### 3.4 Descriptive characterization of cluster profiles

To determine which specific factors significantly differentiate complexity levels, systematic statistical comparisons were conducted across the three identified profiles. One-way analysis of variance (ANOVA) was applied to continuous variables, while Chi-square tests were employed for categorical variables. When statistical significance was achieved (p < 0.05), pairwise comparisons were conducted using Tukey’s Honest Significant Difference (HSD) *post-hoc* test for ANOVA results and Bonferroni correction for Chi-square analyses to control for multiple comparisons and identify specific between-group differences. These analyses revealed statistically significant differences across all measured domains (**Table 2**). To evaluate the relevance of the observed differences between complexity levels, effect sizes were calculated for all significant comparisons (**Table 3**). Cohen’s d was computed for continuous variables, while Cramer’s V was employed for categorical variables, following established guidelines for interpreting effect magnitude (Cohen, 1988). This dual approach enabled both statistical and practical significance to be assessed, ensuring that identified differences were not only statistically reliable but also clinically meaningful.

**Table 2:** Descriptive characteristics and group comparisons among the three profiles.

Characteristic	Low (L) (n*=*100)	Moderate (M) (n*=*87)	High (H) (n*=*83)	Significance test	*Post-hoc*
Age M ± SD	44.66 ± 11.57	24.51 ± 7.14	34.92 ± 10.23	*F =* 96. 14 **	L > H > M
Marital status				χ^2^(2) *=* 87. 23 **	L > H > M (proportion partnered)
Partnered(n)	69	2	35		
Unpartnered (n)	31	85	48		
Education level				χ^2^(4) *=* 13. 22 *	M, H > L (higher attainment in M and H)
Basic*/*secondary (n)	18	35	21		
Higher ed. (n)	72	49	55		
Postgraduate (n)	10	3	7		
Employment status				χ^2^(2) *=* 84. 37 **	L > H > M (employment rate)
Employed (n)	74	8	50		
Unemployed (n)	26	79	33		
Psych. treatment				χ^2^(2) *=* 9. 42 *	M, H > L (treatment history more frequent)
Yes (n)	73	60	73		
No (n)	27	27	10		
Family psychiatric history				χ^2^(2) *=* 1. 86	
Yes (n)	54	52	53		
No (n)	46	35	30		
Gender				χ^2^(2) *=* 8. 85 *	M > L, H (male proportion)
Male (n)	23	37	23		
Female (n)	77	50	60		
Medical comorbidity				χ^2^(2) *=* 118. 23 **	L > H > M (medical comorbidity*)*
Yes (n)	89	47	77		
No (n)	11	40	6		
Childhood maltreatment. M ± SD	42.86 ± 15.33	45.80 ± 13.84	65.95 ± 17.48	*F =* 56. 69 **	H > L, M
Depressive symptoms M ± SD	13.42 ± 5.60	14.02 ± 6.21	22.41 ± 3.17	*F =* 80. 66 **	H > L, M
Personality functioning M ± SD	20.56 ± 8.41	25.13 ± 9.12	36.65 ± 7.67	*F =* 85. 61 **	M, H > L
Emotional dysregulation M ± SD	64.04 ± 15.29	71.02 ± 18.92	95.12 ± 14.11	*F =* 88. 89 **	H> M > L

L, Low-complexity profile; M, Moderate-complexity profile; H, High-complexity profile. Values are presented as mean ± SD for continuous variables and as counts for categorical variables.

*Post-hoc* patterns (e.g., “L > H > M”) indicate significantly higher values in the groups following that order (Bonferroni-corrected p <.05). * p <.05, ** p <.01.

**Table 3:** Global and pairwise effect sizes for categorical and continuous variables across complexity profiles.

Characteristic	Type	Global Effect	L vs M	L vs H	M vs H
Marital status	Categorical	Cramer’s V = 0.36	V = 0.31	V = 0.28	V = 0.19
Employment status	Categorical	Cramer’s V = 0.56	V = 0.47	V = 0.39	V = 0.34
Education level	Categorical	Cramer’s V = 0.22	V = 0.21	V = 0.19	V = 0.11
Gender	Categorical	Cramer’s V = 0.18	V = 0.16	V = 0.09	V = 0.14
Medical comorbidity	Categorical	Cramer’s V = 0.44	V = 0.41	V = 0.33	V = 0.29
Childhood maltreatment (CTQ)	Continuous	η² = 0.30	d = 0.20	d = 1.40	d = 1.28
Depressive symptoms (PHQ-9)	Continuous	η² = 0.38	d = 0.10	d = 1.98	d = 1.70
Personality functioning (OPD-SQS)	Continuous	η² = 0.39	d = 0.52	d = 2.00	d = 1.37
Emotional dysregulation (DERS)	Continuous	η² = 0.40	d = 0.41	d = 2.11	d = 1.44
Age	Continuous	η² = 0.42	d = 2.10	d = 0.89	d = 1.18

Global effect sizes are reported as Cramer’s V for categorical variables and η² for continuous variables. Pairwise effect sizes are reported as Cramer’s V (categorical variables) and Cohen’s d (continuous variables). Pairwise comparisons correspond to statistically significant contrasts following Bonferroni correction.

The Low-complexity profile (L) was characterized by greater sociodemographic stability, including older age, higher rates of partnership, and substantially higher employment. Differences in these variables ranged from moderate to large in magnitude, and this profile also showed the highest prevalence of medical comorbidity. Across clinical and psychosocial indicators, the Low-complexity group consistently displayed the lowest levels of depressive symptoms, childhood maltreatment, emotion dysregulation, and impairment in personality functioning. These contrasts were small to moderate when compared with the Moderate-complexity profile but large to very large when contrasted with the High-complexity group. Overall, this profile showed the most favorable pattern across all domains.

The Moderate-complexity profile (M) was defined by younger age and the lowest levels of partnership and employment, with effect sizes in these sociodemographic variables ranging from moderate to large. It also exhibited the lowest prevalence of medical comorbidity. Across clinical and psychosocial indicators, this group showed values consistently intermediate between the Low- and High-complexity profiles. Differences relative to the Low-complexity group were generally small to moderate, whereas comparisons with the High-complexity profile showed large contrasts. As a whole, the Moderate-complexity profile occupied a clearly intermediate position across all domains.

The High-complexity profile (H) displayed sociodemographic characteristics that were intermediate between the Low and Moderate groups, with moderate-sized differences in age, partnership, and employment. In contrast, this profile exhibited the highest levels of depressive symptoms, childhood maltreatment, emotion dysregulation, and impairment in personality functioning, with large to very large effects relative to both of the other profiles. Medical comorbidity was also elevated in this group, although not to the extent observed in the Low-complexity profile. Taken together, the High-complexity profile was most strongly differentiated by the magnitude of its clinical and psychosocial burden.

### 3.5 Expert assessment of profile interpretability and suggested management strategies

To collect expert opinions, an open survey was conducted among mental health professionals to assess their perceptions of the proposed complexity profiles (low, moderate, and high) and to gather insights on relevant intervention strategies and clinical examples. Sixty-two mental health professionals completed the survey, including 56 psychologists (90.3%) and six psychiatrists (9.7%). Most respondents worked in primary (58%) or secondary (32%) care settings, with clinical experience ranging from two to 25 years (mean = 8 years). Forty-four participants (70%) worked in the public sector, while 18 (30%) were employed in private practice. Forty-nine respondents (80%) primarily provided care for adults, whereas 13 (20%) mainly worked with children or adolescents. Participation in the survey was voluntary and anonymous.

The survey results showed a strong professional consensus regarding the clinical presence of these complexity profiles, with 85% of respondents (n = 53) confirming the relevance of the three distinct complexity profiles (low, moderate, and high) in daily practice. As one experienced psychologist noted: “These profiles capture the reality of what I see in clinical practice - patients don’t fit into simple categories, and we need differentiated approaches” (Psychologist, female, 15 year’s experience). This consensus underscores the practical utility of such classification for guiding clinical decision-making and resource allocation. Only a minority (15%) suggested adjustments to the criteria, primarily to further clarify the boundaries between moderate and high-complexity, particularly regarding the presence of substance use, domestic violence, and suicidality.

Based on the responses, management strategies were recommended for each complexity profile, along with illustrative examples (see **Table 4**).

**Table 4:** Recommended management strategies by the patient’s complexity profile.

Complexity profile	Management strategies	Clinical examples
low-complexity	Brief interventions and group workshops. Periodic follow-up focused on relapse prevention. Referral to community resources and promotion of self-care activities. Use of digital tools for monitoring and support.	*“With lower complexity cases, we can expect more predictable outcomes. The therapeutic alliance forms more easily, and patients are generally more responsive to standard interventions”* (Psychologist, male, 8 years experience).
Moderate-complexity	Individual psychotherapy focused on how to cope with conflicts. Interventions, particularly targeting interpersonal patterns. Coordination with interdisciplinary teams for ongoing follow-up.	*“These patients require more clinical judgment. You need to be prepared to shift your approach based on how they respond. Brief interventions It’s not just about following a protocol anymore”* (Psychiatrist, female, 12 years experience).
High-complexity	Intensive case management, Multidisciplinary team approach (psychiatrist, psychologist, social worker). Crisis planning and risk management protocols. Family/system involvement Supportive interventions based on structured routines and ongoing containment. Frequent clinical supervision, team meetings, and referral to specialized or higher-level services when necessary.Extended treatment timelines	*“High-complexity patients need a team approach. No single provider can address all the needs. We need psychiatric consultation, case management, family involvement, and often longer-term treatment planning”* (Psychologist, female, 20 years experience).

In summary, the survey reveals a high level of acceptance of the complexity profile classification among mental health professionals and provides concrete recommendations for tailored intervention strategies. Strengthening training, coordination, and the use of digital tools are recommended to optimize patient care across all complexity levels.

## 4 Discussion and conclusion

### 4.1 Main findings

This study identified three distinct patient complexity profiles in a treatment-seeking depression population using machine learning methods. The emergence of low, moderate, and high-complexity profiles provides empirical support for the multidimensional heterogeneity that has been increasingly recognized in clinical practice but rarely operationalized systematically. These findings confirm our first hypothesis that qualitatively distinct patterns exist beyond simple diagnostic categorization (46). This aligns with contemporary frameworks proposing that depression is not a single entity but rather a collection of phenotypes with different underlying mechanisms (8, 10, 47, 48).

The three-profile solution supports our second hypothesis regarding significant differences across demographic, clinical, and psychosocial domains. However, our third hypothesis—that higher complexity would reflect accumulation across multiple domains—requires some nuance. The high-complexity profile was not simply characterized by “more of everything.” Instead, it exhibited a particular configuration in which childhood maltreatment, personality dysfunction, and emotional dysregulation converged. This pattern is consistent with developmental psychopathology frameworks (32–34, 36) proposing that early relational trauma disrupts foundational capacities for self-organization and affect modulation, creating enduring vulnerabilities that shape subsequent clinical presentations.

The profiles are distinguished not merely by severity but by the specific interplay of risk mechanisms. Global feature importance rankings identified personality functioning, marital status, depressive symptomatology, emotional dysregulation, employment status, and age as key differentiating factors. Notably, sociodemographic variables emerged as integral components of complexity rather than mere background covariates. This echoes Safford et al.’s (14) Vector Model of Complexity, which emphasizes the interplay of biological, socioeconomic, and behavioral factors as constitutive elements of patient complexity (49–51).

The low-complexity profile demonstrates this multidimensional nature clearly. Despite reporting the highest medical comorbidity, these patients exhibited lower depressive symptoms, better personality functioning, and superior emotional regulation compared to other profiles. This pattern suggests that stable partnerships and employment—both prominent in this group—may buffer against psychological deterioration even in the presence of physical health challenges. This observation aligns with research on social determinants of health (21) demonstrating that relational and occupational stability constitute protective factors that operate independently of medical burden.

The moderate-complexity profile revealed an alternative pathway characterized primarily by sociodemographic instability. Specifically, this group showed the lowest employment rates and highest proportion of unpartnered individuals, combined with relatively preserved personality functioning compared to the high-complexity group. This configuration suggests that complexity can arise through external structural constraints rather than internal psychological vulnerabilities rooted in developmental trauma.

The high-complexity profile exemplifies the developmental cascade described by Gilbert et al. (34) and Li et al. (35), wherein childhood maltreatment establishes vulnerabilities that manifest as personality impairment and emotional dysregulation difficulties. The co-occurrence of elevated childhood trauma exposure (33), markedly impaired personality functioning (25, 26, 29), and pronounced emotional dysregulation (30, 31) in this group reflects the pattern Schaink et al. (7) identified as central to comprehensive mental health care yet rarely operationalized empirically. This profile’s treatment history—more frequent prior psychological interventions despite persistent difficulties—suggests that standard approaches may be insufficient for addressing the deep-seated vulnerabilities characteristic of this presentation.

The strong professional consensus supporting these profiles (85% recognition rate) indicates that complexity profiling resonates with clinicians’ intuitive understanding of patient heterogeneity. As Kaneko et al. (12) observed, practitioners often adapt their interventions to patient complexity regardless of theoretical framework. The convergence between data-driven pattern discovery and expert clinical judgment suggests that machine learning approaches can formalize tacit clinical knowledge into systematic, reproducible frameworks.

### 4.2 Clinical implications

The identification of distinct complexity profiles has several implications for clinical practice and healthcare organization. First, these profiles provide an evidence-based framework for understanding patient heterogeneity beyond symptom severity. This enables recognition of qualitatively different presentations that may require tailored assessment and care planning approaches. This addresses the gap identified by Delgadillo et al. (2) between complexity assessment needs and available systematic methods.

Second, complexity profiling could support treatment stratification in ways that align with contemporary calls for personalized mental health care (6). Expert clinicians recommended differentiated strategies for each profile. Low-complexity patients were deemed appropriate for brief, symptom-focused interventions. Moderate-complexity patients were seen as requiring longer-term support addressing both symptom reduction and psychosocial rehabilitation, particularly around employment and social integration. High-complexity patients were consistently recommended for intensive, trauma-informed psychotherapy addressing personality functioning and emotional regulation alongside symptom management. These recommendations suggest that complexity assessment could guide resource allocation and treatment intensity decisions within stepped-care frameworks.

Third, from a healthcare systems perspective, understanding the prevalence and characteristics of different complexity profiles provides empirical data essential for resource planning and service design. The distribution observed in our sample (32% low, 30.5% moderate, 36.8% high complexity) indicates that a substantial proportion of patients present with high-complexity features. This underscores the need for specialized treatment capacity beyond standard brief interventions.

Importantly, complexity assessment can be performed using routinely collected intake information, demonstrating feasibility for real-world implementation. The six variables accounting for approximately 85% of predictive power—age, personality functioning, marital status, emotional regulation, depressive symptomatology, and employment status—could potentially be streamlined into brief screening instruments suitable for resource-constrained settings.

### 4.3 Limitations

Several limitations warrant consideration. First and most critically, this cross-sectional study did not collect treatment outcome data. Therefore, we cannot assess whether complexity profiles predict differential treatment responses or whether stratified care improves outcomes compared to standard approaches. The clinical utility for treatment planning remains an empirical question requiring prospective investigation.

Second, the sample was drawn from a single outpatient clinic, limiting generalizability across settings, populations, and cultural contexts. The meaning and impact of factors such as partnership status or employment may vary substantially across different societies. Replication in diverse populations is essential before broad implementation.

Third, childhood maltreatment was operationalized through the CTQ total score, aggregating across different types of abuse and neglect. While this approach captures cumulative burden consistent with ACE literature demonstrating dose-response relationships (34), it precludes examining whether specific forms of maltreatment differentially contribute to complexity profiles. Research suggests distinct trauma types may confer different vulnerabilities (32, 33), warranting future investigation with disaggregated subscales.

Fourth, our focus on depression enabled detailed within-diagnosis analysis but leaves unclear whether complexity profiling is diagnosis-specific or more broadly transdiagnostic. Contemporary frameworks propose that processes such as emotional dysregulation and personality dysfunction operate across diagnostic boundaries (30), suggesting complexity assessment might generalize beyond depression.

Fifth, temporal stability of complexity profiles remains unknown. Whether patients remain in the same category across time or whether complexity responds to treatment or life circumstances cannot be determined from cross-sectional data. Longitudinal tracking would illuminate whether profiles represent enduring characteristics or more dynamic states.

Finally, our analytical approach was limited to variables available in our clinical database. Factors not assessed, such as social cognition, metacognitive capacity, or biological markers, might reveal additional complexity dimensions. The interpretability of our profiles assumes that childhood trauma, personality functioning, emotional regulation, and sociodemographic factors adequately capture core dimensions of clinically meaningful heterogeneity. While this assumption is grounded in substantial prior literature, it requires empirical validation.

### 4.4 Future directions

Several research directions emerge as priorities. First, prospective studies examining treatment outcomes across complexity profiles are needed to establish predictive validity. Such studies should test whether complexity-informed treatment matching improves outcomes compared to standard care and whether different profiles respond differentially to specific interventions. Given the treatment recommendations from expert clinicians, particular attention should be paid to whether brief interventions suffice for low-complexity patients while trauma-focused approaches benefit high-complexity presentations.

Second, replication in diverse populations and settings would establish generalizability and enable examination of whether profile structures remain stable across different cultural, healthcare, and socioeconomic contexts. Longitudinal designs tracking complexity over time would illuminate whether profiles represent stable characteristics or dynamic states responsive to intervention, and could identify factors associated with transitions between categories.

Third, expansion to other mental health conditions would evaluate whether complexity profiling is transdiagnostic or diagnosis-specific. If similar profiles emerge across anxiety disorders, trauma-related conditions, and other psychiatric presentations, this would support complexity assessment as a general framework for understanding mental health heterogeneity.

Fourth, development of brief screening instruments for practical implementation represents a pragmatic priority. Abbreviated versions capturing the most discriminating features identified in our analyses could enable rapid complexity screening in busy clinical environments while retaining adequate sensitivity and specificity.

Finally, investigation of developmental pathways through which high-complexity profiles emerge could identify intervention points for prevention. Prospective studies beginning in childhood or adolescence could illuminate how exposure to adversity, combined with other risk factors, crystallizes into the enduring vulnerabilities characteristic of adult high-complexity presentations.

### 4.5 Conclusion

This study provides empirical evidence for distinct, clinically meaningful complexity profiles in treatment-seeking populations with depression. The three identified profiles—characterized by different configurations of sociodemographic stability, clinical severity, developmental adversity, personality functioning, and emotional regulation—align with theoretical frameworks emphasizing depression heterogeneity (8, 10) and the multidimensional nature of patient complexity (7, 14). The findings demonstrate that multidimensional complexity assessment can be performed systematically using routinely available clinical information.

While the cross-sectional design precludes prediction of treatment outcomes, the study establishes descriptive validity and clinical interpretability of complexity-based stratification. The convergence between machine learning pattern discovery and expert clinical recognition suggests this approach captures genuine clinical phenomena rather than statistical artifacts. As Wright and Woods (52) advocate, integrating data-driven methods with clinical expertise bridges the gap between statistical rigor and clinical relevance, supporting movement toward personalized models of psychopathology.

Whether complexity profiling translates into improved clinical outcomes remains to be demonstrated through prospective research. Nevertheless, these findings provide a foundation for systematic complexity assessment that could inform treatment planning, resource allocation, and service design decisions aimed at matching intervention intensity and modality to patient needs within contemporary mental health care systems.

## Data Availability

The raw data supporting the conclusions of this article will be made available by the authors, without undue reservation.

## Supplementary material

Supplementary Figure 1 SHAP importance for low-complexity.

Supplementary Figure 2 SHAP importance for Moderate complexity.

Supplementary Figure 3 SHAP importance for high complexity.

## References

[1] Chen LH Law W Chang DHF Sun D . Editorial: The bio-psycho-social approach to understanding mental disorders. Front Psychol (Internet). (2023) 14:1225433. doi: 10.3389/fpsyg.2023.1225433, PMID: 37397288 PMC10311090

[2] Delgadillo J Huey D Bennett H McMillan D . Case complexity as a guide for psychological treatment selection. J Consulting Clin Psychol. (2017) 85:835–53. doi: 10.1037/ccp0000231, PMID: 28857592

[3] Shippee ND Shah ND May C Mair FS Montori VíctorM . Cumulative complexity: A functional, patient-centered model of patient complexity can improve research and practice. J Clin Epidemiol. (2012) 65(10):1041–51. doi: 10.1016/j.jclinepi.2012.05.005, PMID: 22910536

[4] Grant RW McCloskey J Hatfield M Uratsu C Ralston JD Bayliss E . Use of latent class analysis and k-means clustering to identify complex patient profiles. JAMA Network Open. (2020) 3:e2029068. doi: 10.1001/jamanetworkopen.2020.29068, PMID: 33306116 PMC7733156

[5] Mutai R Sugiyama Y Aoki T Matsushima M . Key characteristics of patient complexity and patient complexity conceptual models/measurement tools: A scoping review. BMJ Open. (2023) 13:e063982. doi: 10.1136/bmjopen-2022-063982, PMID: 37164460 PMC10173976

[6] Maj M Stein DJ Parker G Zimmerman M Fava GA De Hert M . The clinical characterization of the adult patient with depression aimed at personalization of management. World Psychiatry. (2020) 19:269–93. doi: 10.1002/wps.20771, PMID: 32931110 PMC7491646

[7] Schaink AK Kuluski K Lyons Reneé Fortin M Jadad AR Upshur R . A scoping review and thematic classification of patient complexity: offering a unifying framework. J Comorbidity. (2012) 2:1–9. doi: 10.15256/joc.2012.2.15, PMID: 29090137 PMC5556402

[8] Cai N Revez JA Adams MJ Andlauer TFM Breen G Byrne EM . Minimal phenotyping yields genome-wide association signals of low specificity for major depression. Nat Genet. (2020) 52:437–45. doi: 10.1038/s41588-020-0594-5, PMID: 32231276 PMC7906795

[9] Østergaard SD Foldager L . The association between physical illness and major depressive episode in general practice. Acta Psychiatr Scand. (2011) 123:290–6. doi: 10.1111/j.1600-0447.2010.01668.x, PMID: 21219268

[10] Milaneschi Y Lamers F Berk M Penninx BWJH . Depression heterogeneity and its biological underpinnings: Toward immunometabolic depression. Biol Psychiatry. (2020) 88:369–80. doi: 10.1016/j.biopsych.2020.01.014, PMID: 32247527

[11] Nguyen J Kendler KS Chen X Peterson RE Edwards AC . Genetic heterogeneity in major depression: Evidence from subtype analyses. Psychol Med. (2022) 52:3440−3450. doi: 10.1017/S003329172100040X

[12] Kaneko H Hanamoto A Yamamoto-Kataoka S Kataoka Y Aoki T Shirai K . Evaluation of complexity measurement tools for correlations with health-related outcomes, health care costs and impacts on healthcare providers: A scoping review. Int J Environ Res Public Health. (2022) 19(23):16113–16113. doi: 10.3390/ijerph192316113, PMID: 36498188 PMC9741446

[13] Ruscio AM Holohan DR . Applying empirically supported treatments to complex cases: ethical, empirical, and practical considerations. Clin Psychol Sci Pract. (2006) 13(2):146–62. doi: 10.1111/j.1468-2850.2006.00017.x

[14] Safford MM Allison JJ Kiefe CI . Patient complexity: more than comorbidity. The vector model of complexity. J Gen Internal Med. (2007) 22:382–90. doi: 10.1007/s11606-007-0307-0, PMID: 18026806 PMC2219701

[15] Cole MG Dendukuri N . Risk factors for depression among elderly community subjects: a systematic review and meta-analysis. Am J Psychiatry. (2003) 160:1147–56. doi: 10.1176/appi.ajp.160.6.1147, PMID: 12777274

[16] Piccinelli M Wilkinson G . Gender differences in depression. Critical review. Br J Psychiatry. (2000) 177:486–92. doi: 10.1192/bjp.177.6.486, PMID: 11102321

[17] Yan X Huang S Huang C Wu W Qin Y . Marital status and risk for late life depression: a meta-analysis of the published literature. J Int Med Res. (2011) 39:1142–54. doi: 10.1177/147323001103900402, PMID: 21986116

[18] Akhtar−Danesh N Landeen J . Relation between depression and sociodemographic factors. Int J Ment Health Syst. (2007) 1:4. doi: 10.1186/1752-4458-1-4, PMID: 18271976 PMC2241832

[19] Cacioppo J Hughes M Waite L Hawkley L Thisted R . Loneliness as a specific risk factor for depressive symptoms: cross-sectional and longitudinal analyses. Psychol Aging. (2006) 21:140–51. doi: 10.1037/0882-7974.21.1.140, PMID: 16594799

[20] Kessler RC Berglund P Demler O Jin R Koretz D Merikangas KR . The epidemiology of major depressive disorder: Results from the National Comorbidity Survey. JAMA. (2003) 289:3095–105. doi: 10.1001/jama.289.23.3095, PMID: 12813115

[21] Alon N Macrynikola N Jester DJ Keshavan M Reynolds CF Saxena S . Social determinants of mental health in major depressive disorder: Umbrella review of 26 meta-analyses and systematic reviews. Psychiatry Res. (2024) 335:115854. doi: 10.1016/j.psychres.2024.115854, PMID: 38554496

[22] Price R Choi J Vinokur A . Links in the chain of adversity following job loss: how financial strain and loss of personal control lead to depression, impaired functioning, and poor health. J Occup Health Psychol. (2002) 7:302–12. doi: 10.1037//1076-8998.7.4.302, PMID: 12396064

[23] Gabarrell−Pascuet A García-Esquinas E Gracia-García P Lara E Martín-María N Miret M . Data−driven clustering of adults with depression identifies subgroups with distinct sociodemographic and clinical profiles. Psychol Med. (2023) 53:7318−7328. doi: 10.1017/S0033291722004062

[24] Rosenfeld A Benrimoh D Armstrong C Mirchi N Langlois-Therrien T Rollins C . Big data analytics and AI in mental healthcare. arXiv (Cornell University). (2019) 71–137. doi: 10.48550/arxiv.1903.12071

[25] Ehrenthal JC Düx A Baie L Burgmer M . Levels of personality functioning and not depression predict decline of plasma glucose concentration in patients with type 2 diabetes mellitus. Diabetes Res Clin Pract. (2019) 151:106–13. doi: 10.1016/j.diabres.2019.04.011, PMID: 30959148

[26] Ehrenthal JC Kruse J Schmalbach B Dinger U Werner S Schauenburg H . Measuring personality functioning with the 12-item version of the OPD-Structure Questionnaire (OPD-SQS): Reliability, factor structure, validity, and measurement invariance in the general population. Front Psychol. (2023) 14:1248992. doi: 10.3389/fpsyg.2023.1248992, PMID: 37780157 PMC10536238

[27] Zimmermann J Ehrenthal JC Cierpka M Schauenburg H Doering S Benecke C . Assessing the level of structural integration using operationalized psychodynamic diagnosis (OPD): implications for DSM-5. J Pers Assess. (2012) 94:522–3. doi: 10.1080/00223891.2012.700664, PMID: 22808938

[28] Bender DS Skodol AE . Borderline personality as a self-other representational disturbance. J Pers Disord. (2007) 21:500–17. doi: 10.1521/pedi.2007.21.5.500, PMID: 17953503

[29] Newton−Howes G Tyrer P Johnson T . Personality disorder and the outcome of depression: Meta−analysis of published studies. Br J Psychiatry. (2006) 188:13−20. doi: 10.1192/bjp.188.1.13, PMID: 16388064

[30] Aldao A Nolen-Hoeksema S Schweizer S . Emotion-regulation strategies across psychopathology: A meta-analytic review. Clin Psychol Rev. (2010) 30:217–37. doi: 10.1016/j.cpr.2009.11.004, PMID: 20015584

[31] Gratz KL Tull MT Reynolds EK Niec LN Lejuez CW . Multidimensional assessment of emotion regulation and dysregulation in adults. Assessment. (2016) 23:545–59. doi: 10.1177/1073191115577827

[32] Martínez P Gloger S Dagnino P De Medina DD . Early adverse stress and depression severity: A pilot exploration of mediating psychological mechanisms. Dev Psychopathol. (2023) 36(3):1469–78. doi: 10.1017/S0954579423000688, PMID: 37431744

[33] Dagnino P Cordeu C Franco-Chalco E Gloger S Duisallant M Mizon J . The impact of different adverse childhood experiences on the dimensions of emotional dysregulation in adults with major depression. Front Psychol. (2025) 16:1587042. doi: 10.3389/fpsyg.2025.1587042, PMID: 40625435 PMC12231488

[34] Gilbert R Widom CS Browne K Fergusson D Webb E Janson S . Burden and consequences of child maltreatment in high-income countries. Lancet. (2009) 373:68–81. doi: 10.1016/S0140-6736(08)61706-7, PMID: 19056114

[35] Li M D’Arcy C Meng X . Maltreatment in childhood substantially increases the risk of adult depression and anxiety. Psychol Med. (2016) 46:717–30. doi: 10.1017/S0033291715002743, PMID: 26708271

[36] Nanni V Uher R Danese A . Childhood maltreatment predicts unfavorable course of illness and treatment outcome in depression: A meta−analysis. Am J Psychiatry. (2012) 169:141−151. doi: 10.1176/appi.ajp.2011.11020335, PMID: 22420036

[37] Kroenke K Spitzer RL Williams JBW . The PHQ-9: Validity of a brief depression severity measure. J Gen Intern Med. (2001) 16:606–13. doi: 10.1046/j.1525-1497.2001.016009606.x, PMID: 11556941 PMC1495268

[38] Baader T Molina JL Venezian S Rojas C Farías R Fierro-Freixenet C . Validación y utilidad de la encuesta PHQ-9 (Patient Health Questionnaire) en el diagnóstico de depresión en pacientes usuarios de atención primaria en Chile. Rev Chil Neuro-Psiquiatr. (2012) 50:10–22. doi: 10.4067/S0717-92272012000100002

[39] Bernstein DP Fink L Handelsman L Foote J Lovejoy M Wenzel K . Initial reliability and validity of a new retrospective measure of child abuse and neglect. Am J Psychiatry. (1994) 151:1132–6. doi: 10.1176/ajp.151.8.1132, PMID: 8037246

[40] Behn A Guzmán-González M Huepe D Silva JR Arriagada-Vera A Salas G . Validation of the Childhood Trauma Questionnaire Short Form (CTQ-SF) in Chile: Factorial analysis, reliability, convergent and discriminant validity. Child Abuse Negl. (2020) 106:104532. doi: 10.1016/j.chiabu.2020.104532, PMID: 32434060

[41] Gratz KL Roemer L . Multidimensional assessment of emotion regulation and dysregulation: Development, factor structure, and initial validation of the Difficulties in Emotion Regulation Scale. J Psychopathol Behav Assess. (2004) 26:41–54. doi: 10.1023/B:JOBA.0000007455.08539.94

[42] Guzmán-González M Alarcón P Jara Y Rojas G Martínez V Aedo A . Adaptación y validación de la versión en español de la Escala de Dificultades de Regulación Emocional (DERS-E). Univ Psychol. (2014) 13:1065–76. doi: 10.11144/Javeriana.upsy13-3.aave

[43] Ehrenthal JC Dinger U Horsch L Komo-Lange J Grande T Schauenburg H . Validation of the German version of the OPD Structure Questionnaire (OPD-SQ): Clinical and non-clinical samples. Psychother Psychosom Med Psychol. (2019) 69:124–32. doi: 10.1055/a-0719-0268

[44] Franco R Dagnino P . Validation of the OPD Structure Questionnaire Short Form in Chilean clinical samples. (2025).

[45] de la Parra G Undurraga C Crempien C Valdés C Dagnino P Gómez-Barris E . Estructura de Personalidad en Pacientes con Depresión: Adaptación de un instrumento y resultados preliminares. Psykhe. (2018) 27(2). doi: 10.7764/psykhe.27.2.1133

[46] Fried E Nesse RM . Depression is not a consistent syndrome: an investigation of unique symptom patterns in the STAR*D study. J Affect Disord. (2015) 172:96–102. doi: 10.1016/j.jad.2014.10.010, PMID: 25451401 PMC4397113

[47] Lorenzini N de la Parra G Dagnino P Gomez-Barris E Crempien C Ehrenthal JC . Chilean validation of the operationalized psychodynamic diagnosis-structure questionnaire (OPD-SQ) for personality structure. BMC Psychol. (2021) 9:139. doi: 10.1186/s40359-021-00640-4, PMID: 34517907 PMC8436439

[48] Sharma A Verhaak PF McCoy TH Perlis RH Doshi-Velez F . Identifying data-driven subtypes of major depressive disorder with electronic health records. J Affect Disord. (2024) 356:64–70. doi: 10.1016/j.jad.2024.03.162, PMID: 38565338 PMC13464343

[49] Jermy BS Davis KAS Cullen B Ryland H Bebbington P Glanville KP . Associations between depression characteristics and psychiatric comorbidities in UK Biobank. J Affect Disord. (2021) 282:386−393. doi: 10.1016/j.jad.2020.12.133, PMID: 33421867

[50] Kraus C Castrén E Kasper S Lanzenberger R . Serotonin and neuroplasticity—Links between molecular, functional and structural pathophysiology in depression. Neurosci Biobehav Rev. (2017) 77:317−326. doi: 10.1016/j.neubiorev.2017.03.007, PMID: 28342763

[51] Zhu T Mu D Hu Y Cao Y Yuan M Xu J . Association of clinical phenotypes of depression with comorbid conditions, treatment patterns and outcomes: a 10-year region-based cohort study. Transl Psychiatry. (2024) 14:504. doi: 10.1038/s41398-024-03213-2, PMID: 39719438 PMC11668879

[52] Wright AGC Woods WC . Personalized models of psychopathology. Annu Rev Clin Psychol. (2020) 16:389–421. doi: 10.1146/annurev-clinpsy-072319-024803 32070120

