# Supplementary figures and images for: Patient complexity profiles in depression: a machine learning approach to personalized mental health
Source: Front Psychiatry. 2026 Feb 10;17:1741860. doi: 10.3389/fpsyt.2026.1741860 (PMC12930347; doi:10.3389/fpsyt.2026.1741860)

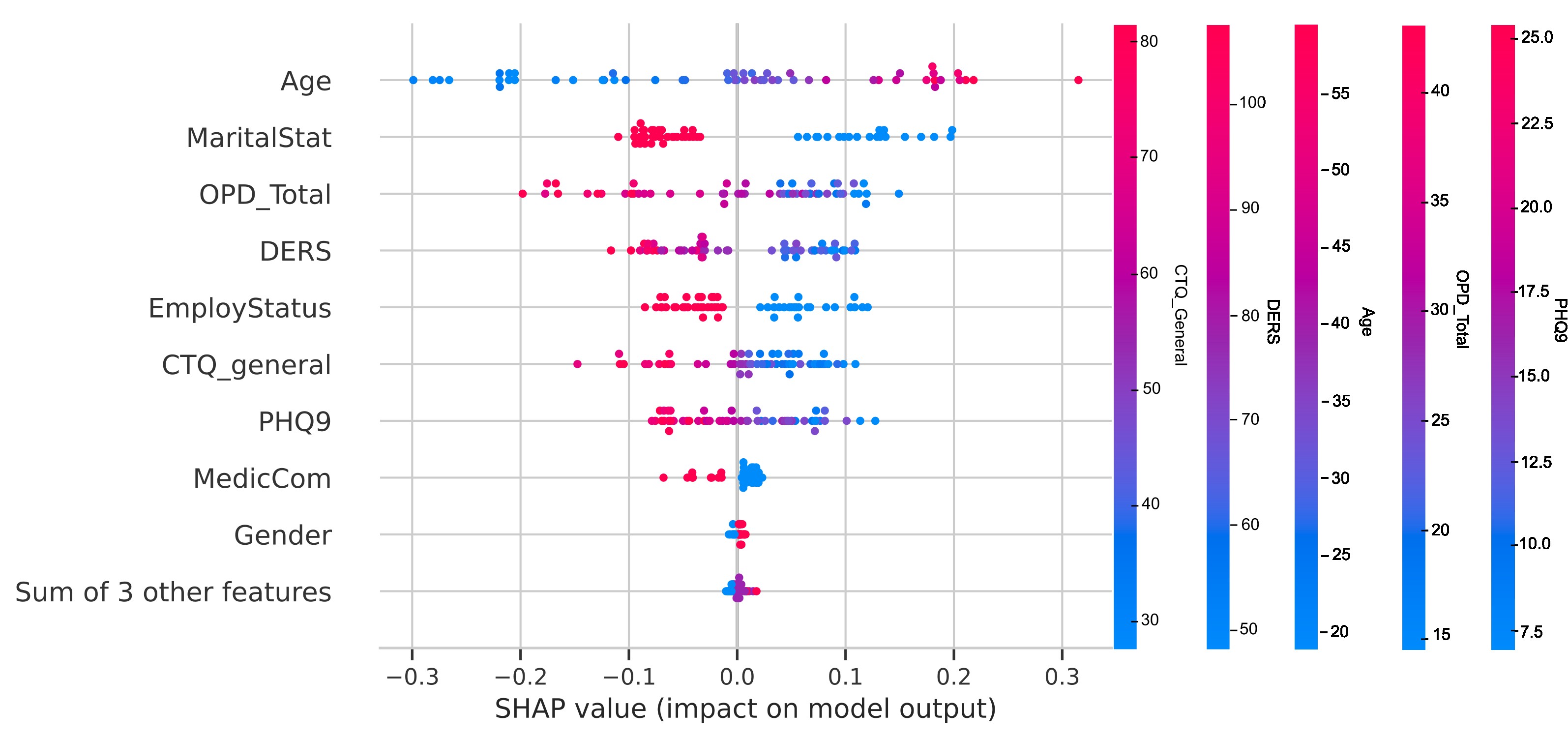

Supplement: Supplementary Figure 1 — SHAP importance for low-complexity. [file Image1.jpeg]

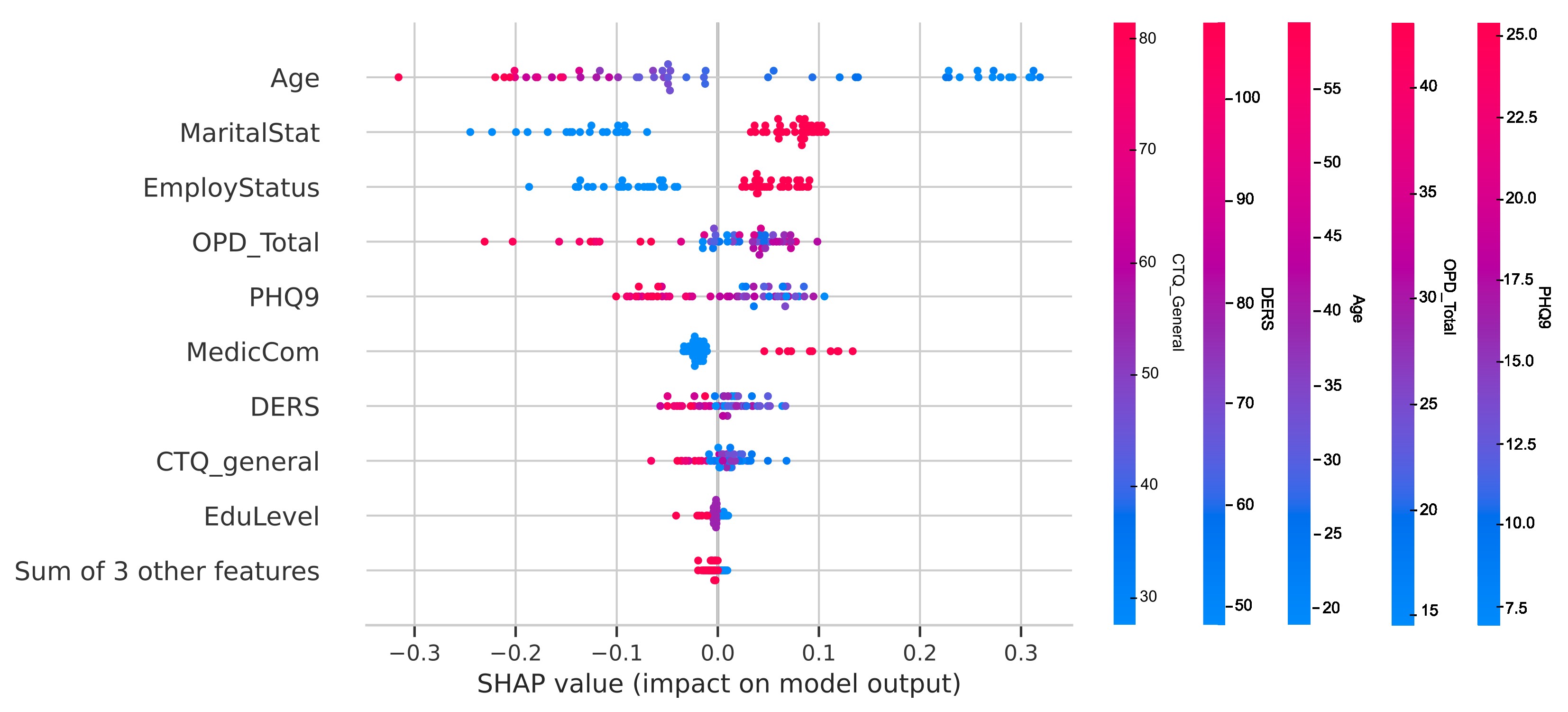

Supplement: Supplementary Figure 2 — SHAP importance for Moderate complexity. [file Image2.jpeg]

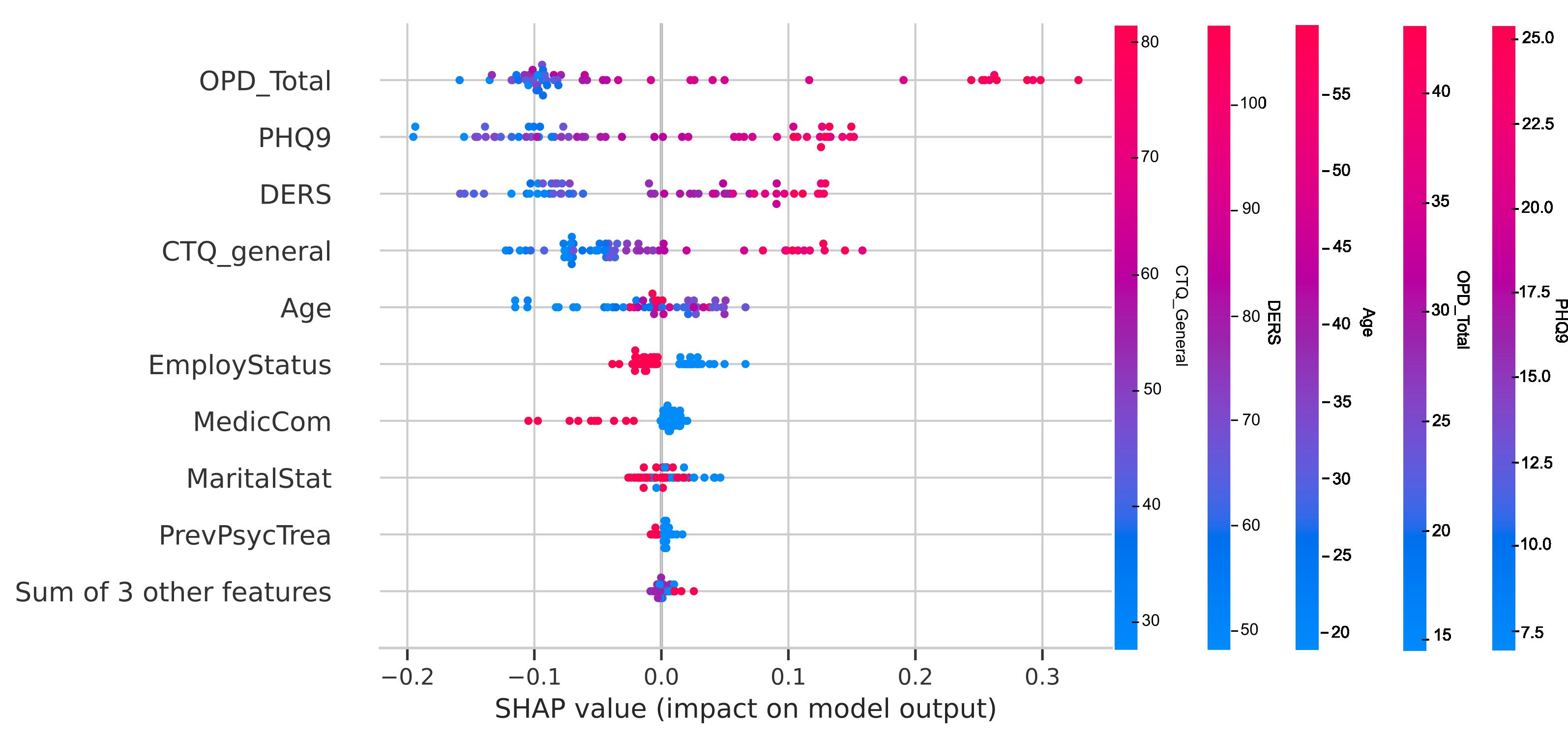

Supplement: Supplementary Figure 3 — SHAP importance for high complexity. [file Image3.jpeg]
